# *Leishmania infantum* in US-Born Dog 

**DOI:** 10.3201/eid2608.200149

**Published:** 2020-08

**Authors:** Marcos E. de Almeida, Dennis R. Spann, Richard S. Bradbury

**Affiliations:** Centers for Disease Control and Prevention, Atlanta, Georgia, USA (M.E. de Almeida, R.S. Bradbury);; Sacramento Area Veterinary Internal Medicine, Sacramento, California, USA (D.R. Spann)

**Keywords:** canine leishmaniasis, Leishmania infantum, vertical transmission, autochthonous leishmaniasis, California, United States, Spain, canis lupus familiaris, leishmaniasis, vector-borne infections, zoonoses, parasitic zoonoses, parasites

## Abstract

Leishmaniasis is a vectorborne disease that can infect humans, dogs, and other mammals. We identified one of its causative agents, *Leishmania infantum*, in a dog born in California, USA, demonstrating potential for autochthonous infections in this country. Our finding bolsters the need for improved leishmaniasis screening practices in the United States.

Leishmaniasis is a tropical and subtropical zoonosis affecting 0.9–1.6 million persons every year. Its manifestations range from self-healing cutaneous lesions to severe visceral leishmaniasis (VL) forms that can be fatal (*1*,*2*). In the Americas, VL is usually caused by *Leishmania infantum* parasites, which several species of blood-feeding sand fly vectors can transmit to humans and other reservoirs. In urban areas, dogs are the main domestic reservoirs of *L. infantum*, maintaining the parasitic life cycle and facilitating transmission of parasites to humans and other mammals (*3*). Alternative routes of *Leishmania* spp. transmission, such as vertical transmission and dog-to-dog transmission by biting, have been associated with autochthonous canine leishmaniasis (Can-VL) (*2*–*4*). In addition, biochemical abnormalities, such as genetic mutations of macrophage proteins, have been associated with increased susceptibility to VL in some breeds, including boxers (*5*).

The accurate differentiation between clinical and subclinical infections is critical in determining the appropriate course of treatment. However, correct diagnosis of Can-VL is challenging because an absence of amastigotes in samples, including blood and tissue, does not rule out infection. Furthermore, the sensitivity and specificity of diagnostic tests vary according to the protocol of the test used (*3*). The geospatial overlap of dogs and vectors infected with *L. infantum* might be linked with human disease. For instance, in focal areas of Brazil the prevalence of infected dogs was associated with the occurrence of clinical VL cases (*8**,**9*). However, in areas to which Can-VL is endemic, attempts to control and prevent Can-VL using controversial procedures, including culling infected dogs, have failed to reduce the spread of human VL cases (*6*,*7*). In North America, most cases of leishmaniasis are acquired during travel or military service in areas to which the disease is endemic. However, leishmaniasis can also be transmitted within the United States. Sylvatic reservoir animals and sand flies, including *Lutzomyia shannoni*, *L. longipalpis*, *L. anthophora*, and *L. diabolica*, are endemic to many US states (*2*). Outbreaks and isolated cases of autochthonous Can-VL affecting foxhounds and other breeds have been reported over the past 2 decades in the United States and Canada (*2*,*3*,*10*). In addition, our laboratory identified a strain of *Leishmania mexicana* in Texas that infected >50 persons, including a patient who shared the same strain with an *L. anthophora* sand fly found in the household (*11*). Here we describe an autochthonous case of Can-VL caused by *L. infantum* sand flies in a US-born dog.

## Case Report

In September 2016, a 1.3-year-old male neutered California-born boxer with no overseas travel history was brought to Sacramento Area Veterinary Internal Medicine (Sacramento, California, USA) by his owner. The dog had granulomatous cutaneous lesions, hypercalcemia, hyperphosphatemia, and hyperglobulinemia, a set of signs that prompted our diagnosis of systemic histiocytosis. The dog was from an apparently healthy litter born to an apparently healthy female relocated from Spain, a country to which Can-VL is endemic. We initially suspected lymphoma but ruled it out on the basis of splenic, lymph node, and bone marrow aspirates and a parathyroid-related peptide test that all yielded negative results. We treated the dog’s cutaneous lesions and biochemical abnormalities with a tapering dosage of oral prednisone. At the next appointment 17 months after the first visit, the dog had a mildly enlarged prescapular lymph node and anemia as well as biochemical abnormalities, including hyperglobulinemia and hypoalbuminemia. He had also lost ≈3.6 kg.

In February 2018, we used light microscopy to identify structures consistent with *Leishmania* spp. amastigotes in an aspirate of the enlarged prescapular lymph node (Figure). We also performed a *Leishmania* indirect immunofluorescence assay serology evaluation with a serial 2-fold dilution of the dog serum. We determined positivity at dilutions of >1:64; the sample tested positive at a titer of 2,048. In March 2018, we confirmed infection with *L. infantum* using PCR followed by DNA sequencing analysis (GenBank accession no. MN991197) as previously described (*12*).

**Figure:**
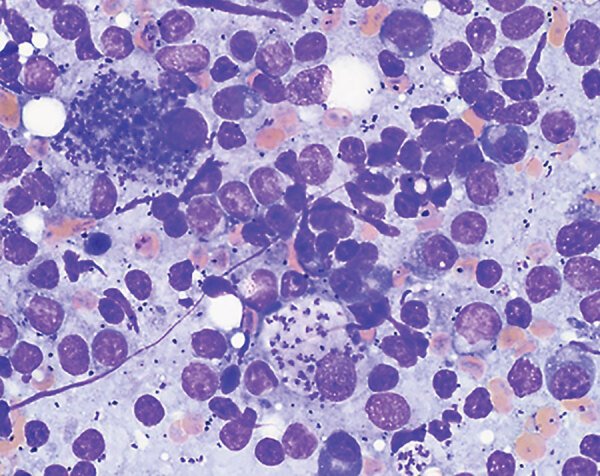
Structures resembling *Leishmania* spp. amastigotes in a lymph node sample from a 1.3-year-old male boxer, California, USA. Sample was prepared in slides stained with Giemsa and examined by light microscopy. Molecular analysis identified the species as *L*. *infantum*. Original magnification ×1,000.

From March through July 2018, we treated the dog with 4 courses of marbofloxacin (100 mg orally every 24 hours) and allopurinol (265 mg orally every 12 hours). In August 2018, we briefly discontinued medications because of the development of neutropenia in the dog. From November 2018 through April 2019, we treated the dog with allopurinol (300 mg every 24 hours), and in April we increased the dose (to 300 mg every 12 hours). In May 2019, strabismus, anisocoria with progressive discomfort, and ataxia developed. A neurologist consult was declined by the dog’s owner, and we resumed marbofloxacin treatment. In mid-May we prescribed antiinflammatory therapy with prednisone as a palliative measure. However, these treatments did not alleviate signs, and we humanely euthanized the dog in late May. We were unable to locate siblings from the same litter for leishmaniasis testing. The breeder reported that the mother died in 2017 of an unknown cause.

## Conclusions

We diagnosed Can-VL in a US-born dog. Given the lack of other risk factors for Can-VL infection, we hypothesize that the dog probably acquired infection through vertical transmission. In countries to which Can-VL is endemic, veterinarians most likely would consider leishmaniasis as a potential diagnosis for fever, granulomatous skin lesions, and weight loss in a dog. However, in the United States, veterinarians often consider leishmaniasis to be a travel-acquired disease and might not suspect this infection in a dog with similar signs. Therefore, veterinarians should consider Can-VL as a potential diagnosis, depending on the dog’s travel and breeding history. Dogs infected with *Leishmania* spp. are important reservoirs, especially in areas where competent vector sand flies are found. Dogs with Can-VL, clinical or subclinical, might contribute to parasite transmission and the occurrence of VL in humans (*7*–*9*,*13*). In areas where sand fly vectors are not prevalent, infectious dogs relocated or returning from areas to which the disease is endemic can still spread Can-VL through transmission routes such as biting, blood transfusion, and breeding (*10*). Current measures to control Can-VL include the regular use of topical sand fly repellents, canine vaccination, and treatment of infected dogs (*6**,**7*).

Changing environmental factors may expand the geographic range of sand fly vectors in North America (*14*), increasing the exposure of humans and animals to the disease. The existence of competent vectors for *Leishmania* spp. in the United States has been demonstrated through a growing number of recently reported autochthonous human cases. For example, >50 human autochthonous cases of cutaneous leishmaniasis caused by *L*. *mexicana* have been reported in Texas and Oklahoma (*11*,*14*). Meanwhile, a possible autochthonous infection with an *Leishmania* *donovani* complex species in a 2-year-old boy was recently reported in North Dakota (*15*).

We hypothesize that this case of Can-VL was probably acquired through vertical transmission because of the lack of evidence supporting California as a leishmaniasis endemic region and the fact that this dog, who had no travel history, was born to a female dog relocated from Spain. Given the lack of surveillance and relative ease of dog-to-dog transmission, Can-VL is probably underreported in North America. The veterinary and public health community should be alert to the existence of autochthonous canine infections and competent vectors of *Leishmania* parasites in the United States, which might contribute to the occurrence of VL in humans. The risk for vertical transmission of Can-VL highlights the need to test all animals either relocated or retuning from areas to which the disease is endemic. This testing will be crucial to improving the surveillance and control of leishmaniasis in North America.
